# Objectively measured cognitive function in insomnia patients with and without comorbid depression treated with cognitive behavioral therapy for insomnia

**DOI:** 10.1186/s12888-025-07460-5

**Published:** 2025-10-02

**Authors:** Sandra Tamm, Susanna Jernelöv, Erik Forsell, Anders Eldh, Adrian Hallek de Oliveira, Liselotte Maurex, Viktor Kaldo, Kerstin Blom

**Affiliations:** 1 Centre for Psychiatry Research, Department of Clinical Neuroscience, Karolinska Institutet, & Stockholm Health Care Services, Region Stockholm, Stockholm, Sweden; 2 Division of psychology, Department of Clinical Neuroscience, Karolinska Institutet, Stockholm, Sweden; 3 Department of Psychology, Faculty of Health and Life Sciences, Linnaeus University, Växjö, Sweden

**Keywords:** Insomnia, depression, CBT-I, CANTAB, cognitive function

## Abstract

**Background:**

Insomnia and depression are common, comorbid conditions with cognitive consequences. Cognitive behavioral therapy for insomnia (CBT-I) improves subjective cognition, but effects on objective performance are unclear. This study aims to examine cognitive differences in insomnia with and without comorbid depression, and changes in cognition following CBT-I.

**Methods:**

This study examined cognitive outcomes in 170 (124 for longitudinal analyses) participants from two randomized clinical trials of internet-delivered 9 or 12 weeks CBT-I for patients with insomnia (*n* = 78) or comorbid insomnia and depression (*n* = 92). Cognitive performance was assessed pre- and post-treatment using selected computerized cognitive tests from the CANTAB battery. Linear regression and mixed-effects models were used for evaluation.

**Results:**

Following CBT-I, improvements were statistically significant on seven out of 17 outcomes across patients with and without comorbid depression: the Rapid Visual Processing task (correct hits, *p* < .001, misses, *p* < .001, latency, *p* = .040), Stockings of Cambridge (problems solved on first choice, *p* = .042), and Affective Go/No gGo (commissions, *p* = .005; omissions, *p* = .022; affective bias, *p* = .020). Comorbid depression was not statistically significantly associated with cognitive performance on most tasks. However, on the Spatial Span task, comorbid depression was associated with lower span length (*p* = .038), fewer attempts (*p* = .021), and longer latency (*p* = .030), suggesting impaired spatial working memory in depressed individuals. No statistically significant associations were found between changes in insomnia or depression severity and changes in cognitive performance after treatment.

**Conclusions:**

Differences in cognitive performance between patients with insomnia and patients with both insomnia and depression seem very small. CBT-I may be associated with improvements in objective cognitive performance across some domains, including attention, working memory, executive function, and emotional processing. Future research should employ better control for e.g. practice effects, and explore any long-term cognitive effects of CBT-I.

**Clinical trials registrations:**

The present study reports follow-up analyses based on data from two previously conducted and preregistered clinical trials (ClinicalTrials.gov Identifier: NCT01663844, study registry date 2012-08-03). The current analyses explore additional outcomes not included in the primary endpoints.

**Supplementary Information:**

The online version contains supplementary material available at 10.1186/s12888-025-07460-5.

## Background

### Insomnia and depression

Insomnia and depression are two of the most common psychiatric conditions, with a point prevalence of between eight and twenty%, and between four and ten%, respectively depending on study population [1–4]. These disorders are highly comorbid; a majority of individuals with depression, as many as around 70%, have comorbid insomnia and around 20% of people with insomnia suffer from depression [1, 5]. Both conditions cause significant suffering for the patient and economic burden for society [6, 7].

Studies have found that insomnia symptoms often emerge before, or simultaneously with, depression [8] and insomnia is a strong predictor for both first and recurrent episodes of depression [9–12]. Cognitive behavioral therapy (CBT) is the first-line treatment for mild to moderate depression as well as insomnia [13–15], and can be provided as internet-delivered CBT with and without therapist support [16, 17]. CBT for insomnia (CBT-I) is a structured, evidence-based psychological treatment designed to address the thoughts, behaviors, and habits that contribute to chronic insomnia and typically includes components such as sleep restriction, stimulus control, cognitive restructuring, sleep hygiene education, and relaxation techniques. Numerous studies have shown a strong effect on insomnia symptoms with smaller effects on objective measures of sleep [12].

Studies in patients with comorbid insomnia and depression have shown that focusing treatment on depression alone is insufficient for many patients suffering from both conditions. The treatment effect is lower, and the insomnia symptoms often remain after treatment, even when the depression is in remission [18, 19]. Conversely, CBT-I can have a significant effect on depression [20], the effect size comparable to CBT for depression [21–23].

### Cognition in insomnia and depression

Patients with insomnia often report cognitive complaints, as shown in observational studies [24]. Experimental studies of sleep deprivation as well as sleep disruption also highlight the clear link between sleep and cognition and have consistently shown negative effects on for example reaction times, sustained attention and memory as a result of lack of sleep [25, 26]. These results do not directly translate to insomnia as insomnia is not necessarily characterized by sleep-deprivation. Instead, insomnia is defined by persistent difficulties falling asleep, maintaining sleep, or waking earlier than desired, accompanied by daytime impairment or distress (e.g., ICD, DSM-5). Although sleep-related worry and subjective perceptions of fragmented or insufficient sleep are common among individuals with insomnia, objective measures such as polysomnography often reveal a discrepancy between perceived and actual sleep duration [27]. A number of studies have therefore compared patients with insomnia with normal sleepers on neuropsychological tests [28–30]. The findings are mixed, likely partially attributed to factors such as small sample sizes, potential effects of confounding variables and unsensitive cognitive tasks [31]. One such confounding factor might be aging, which might affect both sleep pattern, depression and cognition as well as the interplay between such phenomena [32]. Despite some inconsistencies, meta-analyses have pointed towards a deficit in cognitive functions such as episodic memory, problem solving, complex attention, perceptual function and working memory of small to moderate magnitude in insomnia [33] and other studies have also found significant deficits in e.g. attention and episodic memory [31].

Cognitive impairment in depression is a subject that has gained more attention (although with somewhat mixed conclusions). The latest meta-analysis of cognitive function in major depression versus controls [34] showed significant cognitive deficits in episodic memory, executive function, and processing speed, but not for semantic or visuospatial memory, echoing previous studies of the subject [35, 36]. One meta-analysis of brain imaging studies on emotion and cognition in depressed subjects also showed deficits in cognitive control, memory functions and a negative bias in affective information processing [37]. It is not clear whether comorbid insomnia and depression have an additive effect on cognitive function.

Some studies investigated the effect of CBT for depression or insomnia on cognitive function. A meta-analysis of 18 studies including 923 individuals found preliminary evidence for small to moderate effects of CBT-I on subjective measures of cognitive functioning, whereas the effect on objective measures was unclear, due to a small number (*n* = 4) of studies including objective measures [38]. A more recent, large randomized controlled trial (RCT, *N* = 410) comparing digital CBT-I to waitlist similarly found a large effect of CBT-I on self-reported cognitive function but no effect on objective task measures [39]. A small study suggested a positive effect on objective cognitive performance after CBT for depression [40].

In sum, insomnia and depression are two very common, and highly comorbid, disorders that are both often detrimental to the individual and cause large societal costs. They have both been indicated to affect cognitive functions, but how depression and insomnia interact in their effect on cognition is not known. CBT-I has been shown to improve self-reported cognitive impairment in patients with insomnia, but whether such effects translate to objective tests of cognitive function is less clear. Furthermore, it has not been shown whether improvements in insomnia or depression are associated with any positive effects on cognition or whether cognitive function at treatment start predicts treatment outcome.

### Aims

The primary aim of the present study is to address if:

1. Cognitive function in patients with insomnia with or without comorbid depression change after treatment with CBT-I. This aim was pre-registered at Clinical Trials (ClinicalTrials.gov Identifier: NCT01663844, study registry date 2012-08-03).

As secondary aims (not pre-registered), the study aims to investigate the following inquiries:

2. Is there a difference in objective cognitive function, measured with the CANTAB test battery, between insomnia patients with and without comorbid depression?
3. Is any change in cognitive functions related to changes in insomnia symptoms (measured with the Insomnia Severity Index (ISI)) or depressive symptoms (measured with the Montgomery-Åsberg Depression Rating-Scale (MADRS-S))?
4. Does cognitive performance at baseline predict treatment response to CBT-I (i.e. change in insomnia symptoms)?

### Methods

The present study included 170 participants from two RCTs of internet-delivered CBT, a subset of study participants who underwent cognitive testing before and after treatment. Seventy-eight were treated for primary insomnia (henceforth referred to as the Ins-study) and 92 participants were treated for comorbid insomnia and depression (referred to as the Insdep-study), with either a combined insomnia and depression treatment, or a depression treatment with a placebo intervention for insomnia. As reported elsewhere, CBT-I was associated with a significant improvement in insomnia symptoms, see [21, 41].

#### Setting, participants and procedure

The studies were set at the Internet Psychiatry Clinic, a Stockholm Region public health clinic that offers psychologist-guided internet-delivered psychological treatment to adults across Sweden. Adults in the Stockholm region presenting with insomnia, or comorbid insomnia and depression, were eligible for participation. Recruitment was done via the Internet Psychiatry Clinic’s website. Inclusion criteria were as follows: (a) insomnia diagnosis according to DSM-5 criteria, with an ISI (described under Instruments) score > 10; (b) no antidepressant medication or a stable dose for at least two months; (c) no comorbid disorder or substance abuse requiring immediate attention (e.g. suicidality or very severe depression), no disorder considered a contraindication for the treatment (e.g. bipolar disorder or sleep apnea. Individuals presenting with a primary anxiety disorder would likely have been identified and treated accordingly but such diagnoses were not considered a contraindication); (d) not working night shifts; (e) availability and practical ability to participate in the treatment and acceptance of its format (not being away for more than two week during treatment, sufficient knowledge of the Swedish language, regular access to the internet); (f) 18 years or older. Other comorbid disorders were allowed, as was the use of sleep medication.

Participants meeting the DSM-5 criteria for major depression were included in the Insdep-study. Participants without depression were included in the Ins-study. Screening for eligibility was performed through online questionnaires, a brief telephone interview and a face-to-face assessment by a physician or physician plus a psychologist including a structured anamnestic and diagnostic interview (M.I.N.I.) [42]. Participants completed a pre-treatment assessment consisting of on-line questionnaires, sleep diary and actigraphy (presented elsewhere). Subsequently, participants were randomized to nine- (Ins) or twelve- (Insdep) week treatments, with weekly assessments of ISI and MADRS-S. A subgroup of participants completed cognitive testing, described in detail below.

After treatment, participants were asked to complete a post-treatment assessment (the same measurements as the pre-treatment assessment) and come to the clinic for a follow-up assessment by a physician. Those who did not come to the clinic were contacted for a telephone interview. Follow-up measurements were made six months and three years after treatment (no cognitive testing). An overview of the study flows is presented in suppl. Figure 1.

### The Ins-study

The main aim of the study was to develop and test an adaptive treatment strategy using baseline data and data from the first weeks of treatment to predict final outcome, present the prediction to the therapist, and, in the presence of a significant risk of treatment-failure, provide a method to intensify and personalize the treatment to adapt it to the needs of the patient. For a more detailed description of the study, see [41]. The treatment was a nine-week long psychologist-supported internet-delivered CBT-I program. The treatment was highly similar to a previously evaluated treatment program that has shown large and lasting treatment effects that are comparable to traditional face-to-face therapy [43–45]. The main focus of the treatment was sleep restriction and stimulus control, the two primary components of CBT-I [46]. Other components were presented as optional and encompassed facts about sleep, sleep hygiene, cognitive reappraisal, brief acceptance and mindfulness interventions. The treatment consisted of 11 modules delivered over 9 weeks and the full treatment content is presented in suppl. Table 1.

### The Insdep-study

The aim of the study was to compare a combined treatment of evidence-based internet-delivered CBT-interventions for insomnia and depression, to internet-delivered CBT for depression plus a placebo intervention for insomnia. Both treatments were therapist-supported, i.e. consisted of online material supported by regular contact (mainly through asynchronous messages within the digital treatment platform) with psychologists at the Internet Psychiatry Clinic. For a detailed description please see [21].

#### Combined insomnia and depression treatment

The main treatment components were the insomnia interventions sleep restriction or bed-time consistency followed by sleep compression, and the depression intervention behavioral activation. Other interventions were oriented towards both sleep and mood: facts about sleep and depression, cognitive reappraisal, problem solving, brief acceptance and mindfulness interventions. The treatment consisted of 9 modules delivered over 12 weeks and the full content is presented in suppl. Table 2. The techniques and focus of the CBT-I part were comparable to the CBT-I treatment in the Ins-study.

#### Depression treatment with a placebo insomnia intervention

The intervention consisted of the standard internet-delivered CBT-treatment for depression offered at the Internet Psychiatry Clinic [47], with the addition of a placebo-intervention for insomnia. The purpose of this intervention was to give the control group the best available treatment for depression, and at the same time create credibility for the treatment’s effect on insomnia symptoms. The placebo-intervention is a desensibilization treatment that has been previously used for a similar purpose [48]. For analyses concerning baseline differences, all patients were included in analyses. For analyses related to treatment with CBT-I, from this study we only analyzed data from the 46 participants receiving CBT-I in combination with depression treatment.

### Measures/outcomes

#### Cognitive tests

In order to assess cognitive function, a subset of tests from the widely used and validated Cambridge Neuropsychological Test Assessment Battery (CANTAB) was used (Cambridge Cognition, 2019). The first test session was scheduled within two weeks before treatment start. The second testing was similarly booked in close proximity to the end of treatment. The cognitive tests took place at the Internet Psychiatry Clinic in Stockholm, during office hours. A test administrator was present during the entirety of the testing. The administrator gave scipted instructions (translated from English to Swedish) and answered potential questions. The test administrator was not blinded to treatment-group (Ins or Insdep).

#### Cambridge neuropsychological test automated battery (CANTAB)

The CANTAB research suite 6 includes 25 computer based cognitive tests assessing different aspects of cognitive function [49]. CANTAB was originally developed to assess cognitive function in elderly and people suffering from dementia. The tests have been standardized using a large sample of healthy individuals [50] and have been validated in studies of patients with specific brain lesions [51]. Since then, CANTAB has also been utilized in populations with different psychiatric disorders, e.g. depression [52]. Depending on the sampled population, the tests can be combined into batteries. The test battery used in this study was a battery originally composed for measuring cognitive aspects of mood disorders. The tests used were primarily chosen for their documented sensitivity to cognitive change in affective conditions [53–55]. Examples of studies using the tests are: [56] (Delayed matching to sample) [57], Rapid Visual Information Processing) [52], (One Touch Stockings of Cambridge) [56] and [58], (Spatial Span), and [59] (Affective Go/No-go). However, there is no dedicated test battery in CANTAB for measuring cognitive functions in insomnia, and, to our knowledge, this battery has not been utilized previously in studies of insomnia. The tests were administered using a touch screen PC of the brand TabletKiosk (Windows XP Professional, Intel ^®^ Celeron ^®^ M CPU 423 @ 1,06 GHZ, 0,99 GB RAM), specifically produced for this purpose by Cambridge Cognition Ltd. For a more exhaustive description of CANTAB, see https://cambridgecognition.com/digital-cognitive-assessments/. The following tests were administered, with a total test time of around 60 min, described in the order of administration:

##### Motor screening (MOT)

MOT is a training procedure designed to relax the subject and to serve as an introduction to the test computer and touch screen. Moreover, MOT screens for difficulties with vision, movement or comprehension. A series of crosses is shown in different locations on the screen. After a demonstration of the correct way to point – using the forefinger of the dominant hand, the subject is instructed to touch the crosses in turn as they flash pink and green on the screen. The administration time of the test is around one minute (Cambridge Cognition, 2014). Baseline data from this task are presented for reference.

##### Delayed matching to sample (DMS)

DMS measures visual recognition memory by testing simultaneous and delayed matching to sample. It is described to be primarily sensitive to deficits in the medial temporal lobe area, with some input from the frontal lobes. The subject is shown a complex visual pattern and then, after a brief delay, four patterns. Each pattern is made up of four sub-elements, each of a different color. One of the patterns displayed is identical to the sample pattern displayed first. Out of the other three, one is a novel distractor pattern, one has the shape of the sample pattern and the colors of the distractor, and one has the colors of the sample pattern and the shape of the distractor. However, all four patterns have one quadrant in common with the sample. The subject is instructed to choose the pattern that matches the sample. The sample and choice patterns are either shown simultaneously, or there is a delay between covering the sample pattern and showing the four choice alternatives. The delay is either zero, four or twelve seconds long. If an incorrect choice is made, the subject must continue making choices until the correct pattern has been chosen. In the test mode used, there are three practice trials, followed by 20 counterbalanced test trials, comprised of five simultaneous and five at each of the three time intervals. The subject is offered a pause for rest, after which there are 20 more counterbalanced test trials, making a total of 40.

The outcome measures used were mean latency and number of correct responses. The test takes approximately twelve minutes to administer.

##### Rapid Visual Information Processing (RVP)

RVP is a test of visual sustained attention. The test has previously been shown to be sensitive to dysfunction in the parietal and frontal lobe areas of the brain as well as a sensitive measure of general performance (Cambridge Cognition, 2014) [54]. Digits ranging from two to nine appear in a white box in the center of the screen. The digits appear in a pseudo-random order, at the rate of 100 digits per minute. Subjects are tasked to detect target sequences of digits by using the press pad. When one of the target sequences appear, the subject is instructed to press the pad as quickly as possible Target sequences occur at the rate of 16 every two minutes. The test consists of a “warm up” practice phase which lasts for two minutes and is not scored, followed by a test phase. Total administration time is around six minutes and thirty seconds. The test mode used contains three target digit sequences: 2-4-6, 4-6-8 and 3-5-7. There are 27 target sequences in total during the assessed blocks (i.e. in the test phase).

The outcome measures used were total number of correct hits, number of misses, number of false positives and mean latency, i.e. the mean response latency for correct hits.

##### One touch stockings of Cambridge (OTS)

OTS measures frontal lobe function, including working memory, executive function, and planning (Cambridge Cognition, 2014). The subject is shown two displays, containing three colored balls each, one at the top and one at the bottom half of the screen. The graphics on the displays make it appear like the balls are held in three stockings or socks hanging next to each other. The left “sock” has room for three balls, the middle one two balls and the right one has room for one. The three balls are placed in a different pattern in the two displays. In the mode used, there are six boxes at the bottom of the screen, numbered one to six. The subject is tasked with calculating how many times you would have to move the balls in bottom display to match the pattern of the top display and then chose the correct number out of the boxes at the bottom of the screen. The maximum number of moves required are five, i.e. one fewer than the number of boxes at the bottom of the screen. This is to prevent subjects from automatically assuming the largest number of moves to solve the problems that seem the most difficult. If an incorrect choice is made, the subject must continue making choices until the correct number of moves have been identified.

The outcome measures used were mean number of problems solved on first choice, mean choices to correct and mean latency of correct answers. The test takes around ten minutes to administer.

##### Spatial Span (SSP)

SSP assesses working memory capacity and is a computerized version of the Corsi Blocks test [60]. It is designed to give a measure of frontal lobe functioning (Cambridge Cognition, 2014). The test screen shows a pattern of white boxes. During a test sequence some of the boxes change in color, one by one, in a variable sequence. At the end of every sequence, a tone indicates the subject should touch each of the boxes that where colored, in the same order as they were previously presented. The sequence and color used change between sequences to minimize interference. The number of boxes in the sequence starts at two and increases to a final level of nine. There are three possible sequences at each level. As soon as the subject passes a sequence they will immediately progress to the next level. If a subject fails each of the three possible sequences at any level, the test terminates.

The outcome measures used were span length, i.e., the number of boxes included in the longest sequence that the participant reproduced correctly, number of incorrect responses, number of attempts, as well as choice latency. The test takes around five minutes to administer.

##### Affective Go/No-go (AGN)

The AGN test measures affective information processing. A series of words is rapidly presented in the center of the screen. Words are displayed one at a time. Each word is displayed for 300 milliseconds. There is an interval between the words of 900 milliseconds. In the test mode used, the words fall into either positive (Pos) or negative (Neg) valence. The subject is given a target valance (Pos or Neg) and is asked to press a button when a word that matches this valance is presented. The test mode consists of ten 18-word blocks, where the first two are practice blocks that are not scored. The blocks were presented in the following order, named after the target valence: Neg, Neg, Pos, Pos, Neg, Neg, Pos, Pos, Neg, Neg.

The outcome measures used were latency to correct response, false positives (commissions), misses (omissions) and affective bias. The test takes around six and a half minutes to administer.

Before the start of this study, the AGN was translated from English to Swedish. Swedish words of comparable relative frequencies (i.e., how commonly used they are in the Swedish language), word length and affective valence were determined. Relative frequencies were calculated using the corpus infrastructure of the Swedish Language Bank [61]. Words of comparable affective valence were determined by a reference group of 15 people chosen through convenience selection. The group assessed 240 selected words on a seven grade likert-scale, ranging from very negative to very positive.

#### The Swedish National adult reading test (NART-SWE)

In addition to the CANTAB-tests, a swedish version of the brittish *National Adult Reading Test* (NART) [62], called NART-SWE, was administered. The instrument is designed to measure pre-morbid cognitive ability (intelligence). The subject is tasked with reading aloud a list of 50 unusual words and scores one point for every correct pronounciation. The test result is then converted into an estimate of the subjects general premorbid intelligence.

#### Insomnia severity index (ISI)

The ISI is a self-report measure assessing subjective severity of insomnia symptoms, i.e. sleep onset and sleep maintenance difficulties (both during the night and early morning awakenings) as well as perceived interference on daily functioning, the level of satisfaction with one’s current sleep pattern, noticeability of impairment attributed to the sleep problems and the level of concern caused by the sleep problems. The ISI has seven items and a total score of between zero and 28 [63]. It has displayed good validity and reliability in identifying and defining the level of severity of insomnia in comparison to other methods such as sleep diaries, polysomnography data and clinical assessment [63]. Several studies have also shown that ISI has sufficient sensitivity to efficiently measure treatment outcome of insomnia treatment in research as well as in a clinical population [63, 64]. Furthermore, ISI has been validated for online-administration [65].

#### Montgomery Åsberg depression rating scale- self assessment (MADRS-S)

The MADRS-S is a self-report measure assessing severity of depression symptoms during the past three days. It is designed to be sensitive to change in symptoms resulting from antidepressant therapy. It consists of nine items: mood, feelings of unease, sleep, appetite, ability to concentrate, initiative, emotional involvement, pessimism, and zest for life. Each item is answered on a likert-scale from zero to six, with a higher number indicating more severe symptoms. The sum of the answers makes a total score of between zero and 54. The scale has been demonstrated to have good reliability and strongly correlate with clinician administered MADRS, as well as to the widely used Beck Depression Inventory [66, 67].

### Statistical analyses

All statistical analyses were conducted using R (version 4.2.2). To investigate the association between baseline depression diagnosis and cognitive outcomes, a series of linear regression models were estimated using the lm() function. Analyses were restricted to complete cases, and results are reported with corresponding 95% confidence intervals and p-values. Three linear mixed effects models were specified to evaluate this association. The first model (crude) included depression diagnosis as the sole dichotomous predictor of the cognitive outcome (depression diagnosis = 1, no depression diagnosis = 0). The second model (partly adjusted) incorporated additional demographic and cognitive covariates, specifically age, sex, education, and NART verbal IQ. The third model (fully adjusted) further accounted for baseline ISI scores and current use of sleep medication (as a dichotomous variable). For each model, the regression coefficient for the depression variable, along with its 95% confidence interval (estimated using the confint() function) and *p*-value (from the model summary), was extracted and reported.

To assess changes in cognitive outcomes following treatment with CBT-I, a linear mixed-effects model was fitted using the lme() function from the nlme package (version 3.1). The model included session (pre/post) and study as fixed effects, with a random intercept specified for each participant to account for repeated measures and intra-individual variability. The model formula was specified as outcome ~ Session + Study, with random effects defined as ~ 1 | ID. Model estimation was performed using restricted maximum likelihood (REML), and missing data were addressed using the na.exclude option. Fixed-effect estimates and their 95% confidence intervals were obtained using the intervals() function. Statistical significance of fixed effects was evaluated using marginal Type III ANOVA as implemented in the anova() function. To quantify the magnitude of the session effect, Cohen’s *d* was calculated for the outcome variable across sessions.

To examine the relationship between changes in depression and insomnia symptoms and change in cognitive outcomes, separate linear regression models were estimated. One model examined the association between change in the MADRS-S score (MADRS-S_prepost_change) and change in the cognitive outcome, while another assessed the association between change in ISI score (ISI_prepost_change) and change in the same outcome. Each model included estimates of the unstandardized regression coefficient and its 95% confidence interval, the standardized regression coefficient (β), and the *p*-value assessing the statistical significance of the predictor. Both models were estimated using the lm() function with the na.exclude option to handle missing data by excluding rows with missing values in either the predictor or the outcome.

Lastly, to explore the association between baseline cognitive variables and changes in ISI score, a series of linear regression models were constructed. The first model (crude) included only the cognitive predictor. The second model (partly adjusted) included additional covariates: depression status (yes/no), age, sex, education, and NART. The third model (fully adjusted) further included baseline ISI scores and current use of sleep medication. All models were estimated using the lm() function with the na.exclude option, and results included the regression coefficient, 95% confidence interval, and p-value for each predictor of interest.

All analysis code can be downloaded from: 10.5281/zenodo.17161599.

## Results

### Sample characteristics

Sample characteristics are shown in Table 1. Baseline values for cognitive outcomes are shown in Table 2. A detailed list of antidepressants and sleep medication reported at baseline is presented in suppl. Table 3.

**Table 1:** Sample characteristics. Numerical variables given in mean (sd), categorical variables as n with (%). Insdep = Insomnia and depression study. Ins = Insomnia study with adaptive treatment. MADRS = Montgomery Åsberg depression rating Scale, ISI = Insomnia severity Index, NART = National adult reading test

Variable	Insdep (All)	Insdep (CBT-I sample)	Ins
*n*	92	46	78
Age	42.0 (12.3)	41.3 (12.6)	46.7 (13.2)
Sex (females)	57 (62.0%)	32 (69.6%)	53 (67.9%)
Education			
Primary school	5 (5.5%)	4 (8.9%)	2 (2.6%)
High School	52 (57.1%)	26 (57.8%)	16 (20.5%)
University	34 (37.4%)	15 (33.3%)	60 (76.9%)
Working/Studying (yes)	73 (80.2%)	38 (84.4%)	65 (83.3%)
Current sleep medication (yes)	37 (40.7%))	19 (41.3%)	48 (62.3%)
MADRS sum pre (mean (sd))	24.2 (6.3)	24.2 (5.9)	13.0 (5.3), NA = 2
MADRS sum post (mean (sd))	17.6 (8.2), NA = 10	17.9 (7.0), NA = 6	8.1 (5.3), NA = 1
ISI sum pre (mean (sd))	18.7 (3.7)	19.0 (3.2)	16.9 (4.2), NA = 2
ISI sum post (mean (sd))	12.9 (4.8), NA = 9	12.0 (4.8), NA = 5	7.9 (4.6), NA = 5
NART raw scores	39.4	39.0 (6.2), NA = 2	39.2 (6.75), NA = 1

**Table 2:** Baseline values and post treatment values for cognitive outcomes (*N* = 124). Insdep = Insomnia and depression study. Ins = Insomnia study with adaptive treatment MOT = motor screening, DMS = Delayed matching to Sample, RVP = Rapid visual Processing, OTS = One touch stockings of Cambridge, SSP = Spatial span Task, AGN = Affective go/No go

	Pre		Post	
Study	Insdep (*N* = 46)	Ins (*N* = 78)	Insdep (*N* = 46)	Ins (*N* = 78)
variable				
MOT				
Mean latency	1003.60 (268.97)	915.28 (218.77)	995.89 (253.55)	879.28 (215.32)
Number of correct responses	9.72 (1.50)	9.99 (0.11)	8.80 (3.22)	9.84 (1.28)
DMS				
Mean latency	3200.78 (743.17)	3353.24 (796.93)	3088.34 (877.66)	3238.17 (831.37)
Number of correct responses	35.22 (3.73)	36.29 (2.58)	36.42 (2.59)	36.19 (5.15)
RVP				
Correct hits	39.43 (7.54)	39.69 (5.77)	43.26 (7.74)	43.16 (7.82)
Number of misses	9.76 (5.37)	9.54 (4.21)	6.42 (4.67)	6.82 (4.42)
False positives	1.22 (1.52)	1.35 (2.23)	1.32 (1.85)	1.32 (2.37)
Mean latency	407.39 (79.62)	424.15 (82.57)	413.99 (80.61)	393.61 (63.60)
OTS				
Number of problems solved on first choice	17.04 (2.94)	17.06 (2.10)	17.87 (1.76)	17.56 (2.17)
Mean choices to correct	1.17 (0.15)	1.18 (0.15)	1.13 (0.13)	1.16 (0.17)
Mean latency	11796.57 (9998.55)	12367.44 (7078.62)	9426.39 (7546.00)	11448.92 (7906.04)
SSP				
Span length	6.50 (1.44)	6.63 (1.23)	6.13 (1.80)	6.84 (1.40)
Errors	13.50 (5.98)	15.04 (6.12)	13.90 (6.88)	13.23 (6.00)
Number of attempts	9.35 (2.18)	9.88 (1.96)	9.10 (2.60)	9.65 (1.98)
Latency	9461.69 (2913.35)	8636.09 (2183.13)	8422.07 (2333.17)	8654.04 (2605.03)
AGN				
Latency	508.32 (69.27)	522.74 (62.31)	522.34 (57.12)	517.71 (59.77)
Commissions	6.76 (4.69)	6.28 (4.87)	4.52 (3.32)	6.00 (4.82)
Omissions	6.91 (10.38)	3.83 (5.00)	3.48 (7.08)	3.53 (5.19)
Affective bias	−24.83 (31.98)	−12.85 (32.16)	−9.23 (31.66)	−5.95 (26.17)

### Difference in objective cognitive function between insomnia patients with and without comorbid depression

Differences in cognitive function between insomnia patients with and without depression was investigated using linear regression and are presented in Table 3.

**Table 3:** Depression vs. not at baseline (*N* = 170). Insdep = Insomnia and depression study. Ins = Insomnia study with adaptive treatment. DMS = Delayed matching to Sample, RVP = Rapid visual Processing, OTS = One touch stockings of Cambridge, SSP = Spatial span Task, AGN = Affective go/No go

Variable	Crude model		Partly adjusted model^1^		Fully adjusted model^2^	
	Estimate (95% CI)	p	Estimate (95% CI)	p	Estimate (95% CI)	p
DMS						
Number of correct responses	−0.958 (−1.85–0.07)	0.035	−0.931 (−1.9-0.04)	0.06	−0.964 (−2.01-0.08)	0.071
Mean latency	−81.657 (−319.68-156.37)	0.499	−12.527 (−272.11-247.06)	0.924	41.048 (−238.9-320.99)	0.772
RVP						
Correct hits	−0.301 (−2.33-1.73)	0.77	−1.108 (−3.26-1.05)	0.311	−1.564 (−3.87-0.74)	0.181
Number of misses	0.418 (−1.03-1.86)	0.569	0.798 (−0.73-2.33)	0.305	1.299 (−0.34-2.93)	0.119
False positives	0.143 (−0.47-0.76)	0.646	0.13 (−0.53-0.79)	0.699	0.086 (−0.63-0.8)	0.811
Mean latency	−25.441 (−49.34–1.54)	0.037	−14.311 (−40.68-12.05)	0.285	−10.804 (−38.75-17.15)	0.446
OTS						
Number of problems solved on first choice	−0.12 (−0.83-0.59)	0.741	−0.415 (−1.2-0.37)	0.296	−0.309 (−1.14-0.52)	0.463
Mean choices to correct	0.008 (−0.04-0.05)	0.73	0.023 (−0.03-0.07)	0.374	0.017 (−0.04-0.07)	0.53
Mean latency	−988.004 (−3360.57-1384.57)	0.412	667.719 (−1916.44-3251.88)	0.611	258.385 (−2512.97-3029.74)	0.854
SSP						
Span length	−0.161 (−0.56-0.24)	0.424	−0.474 (−0.86–0.09)	0.017	−0.442 (−0.86–0.02)	0.038
Number of attempts	−0.559 (−1.17-0.05)	0.072	−0.978 (−1.62–0.34)	0.003	−0.811 (−1.5–0.12)	0.021
Errors	−1.397 (−3.2-0.4)	0.127	−1.755 (−3.76-0.25)	0.086	−1.164 (−3.33-1)	0.289
Latency	794.157 (6.66-1581.66)	0.048	806.954 (−71.05-1684.96)	0.071	1043.747 (100.14-1987.35)	0.03
AGN						
Commissions	0.566 (−0.92-2.05)	0.454	0.127 (−1.53-1.78)	0.88	0.114 (−1.68-1.91)	0.9
Omissions	2.004 (−0.12-4.13)	0.064	1.233 (−1.1-3.56)	0.297	1.064 (−1.46-3.59)	0.406
Latency	−8.691 (−28.76-11.38)	0.394	3.362 (−18.21-24.93)	0.759	2.755 (−20.23-25.74)	0.813
Affective response bias	−5.748 (−15.81-4.32)	0.261	−8.799 (−19.92-2.32)	0.12	−9.401 (−21.34-2.54)	0.122

^1^Adjusted for Age, Sex, Education, and NART

^2^Adjusted Age, Sex, Education, and NART, ISI score and use of sleep medication

For the Delayed Matching to Sample (DMS) task, there was no association for depression and number of correct responses (β = −0.964, 95% CI: −2.01 to −0.08, *p* =.07) or mean latency (β = 41.048, 95% CI: −238.9 to 320.99, *p* =.77) in fully adjusted models.

For Rapid Visual Information Processing (RVP), there was no significant association for depression and correct hits (β = −1.564, 95% CI: −3.87 to 0.74, *p* =.18), number of misses (β = 1.299, 95% CI: −0.34 to 2.93, *p* =.12), false positives (β = 0.086, 95% CI: −0.63 to 0.80, *p* =.81) or mean latency (β = −1384.57, 95% CI: −1916.44 to 3251.88, *p* =.41) in fully adjusted models.

For One Touch Stockings of Cambridge (OTS), there was no association for depression and number of problems solved on first choice (β = −0.309, 95% CI: −1.14 to 0.52, *p* =.46), mean choices to correct (β = 0.017, 95% CI: −0.04 to 0.07, *p* =.53) or mean latency (β = 41.048, 95% CI: −238.9 to 320.99, *p* =.77) in fully adjusted models.

For Spatial Span (SSP), there was a negative association for depression and span length (β = −0.442, 95% CI: −0.86 to −0.02, *p* =.04) and number of attempts (β = −0.811, 95% CI: −1.5 to −0.12, *p* =.02), and a positive association for latency (β = 1043.747, 95% CI: 100.14 to 1987.35, *p* =.03), whereas no association was found for errors (β = −1.164, 95% CI: −3.33 to 1.0, *p* =.29) in fully adjusted models.

For Affective Go/No-go (AGN), there was no significant association for depression and commissions (β = 0.114, 95% CI: −1.68 to 1.91, *p* =.90), omissions (β = 1.064, 95% CI: −1.46 to 3.59, *p* =.41), latency (β = 2.755, 95% CI: −20.23 to 25.74, *p* =.81) or affective bias (β = −9.401, 95% CI −21.34 to 2.54, *p* =.12) in fully adjusted models.

### Change in cognitive function after treatment with CBT-I

**Fig. 1:**
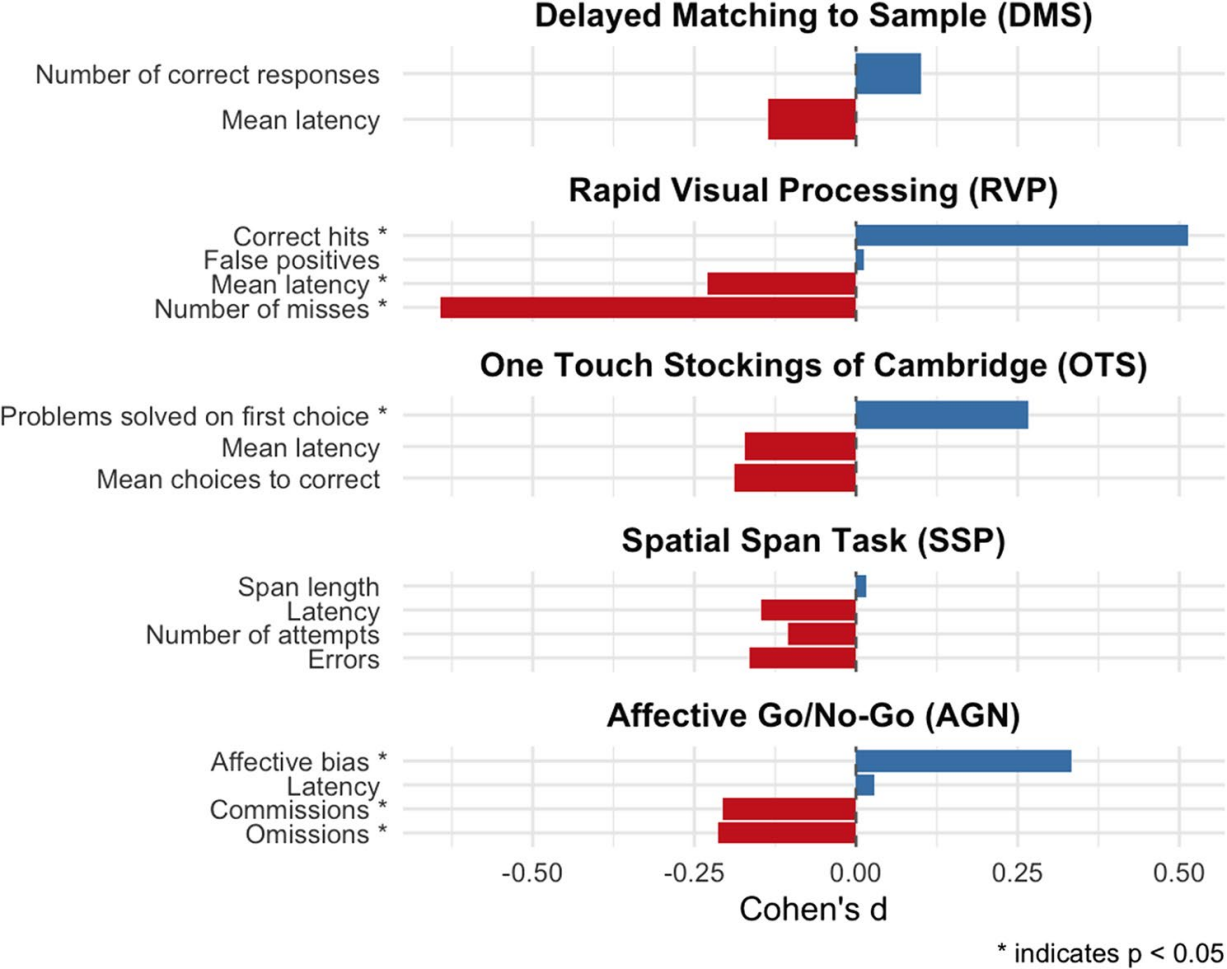
Standardized withing group effect sizes (Cohen’s d) for the changes from pre to post treatment on the different cognitive tasks

Change in cognitive function after CBT-I was investigated using mixed effects models. Pre and post values for the outcomes are presented in Table 2 and the results from the analyses are presented in Table 4; Fig. 1. Individual data as well as study means are plotted in suppl. Figure 2–4.

**Table 4:** Change in cognitive function from pre to post treatment with CBT-I (*N* = 124). Estimates correspond to the actual mean change on the variable derived from a mixed effects model. DMS = Delayed matching to Sample, RVP = Rapid visual Processing, OTS = One touch stockings of Cambridge, SSP = Spatial span Task, AGN = Affective go/No go

Variable	Estimate (95% CI)	*p*	Cohen’s d
DMS			
Number of correct responses	0.33 (−0.64-1.31)	0.498	0.101
Mean latency	−97.66 (−221.98-26.66)	0.122	−0.136
RVP			
Correct hits	3.18 (1.82–4.55)	0	0.513
Number of misses	−2.61 (−3.43–1.79)	0	−0.643
False positives	0.08 (−0.41-0.57)	0.741	0.012
Mean latency	−13.54 (−26.48–0.6)	0.04	−0.23
OTS			
Number of problems solved on first choice	0.45 (0.02–0.88)	0.042	0.266
Mean choices to correct	−0.02 (−0.05-0.01)	0.124	−0.189
Mean latency	−1678.04 (−3445.95-89.88)	0.063	−0.172
SSP			
Span length	−0.01 (−0.31-0.29)	0.953	0.015
Number of attempts	−0.26 (−0.72-0.21)	0.28	−0.106
Errors	−1.04 (−2.69-0.62)	0.216	−0.165
Latency	−328.06 (−878.33-222.22)	0.239	−0.147
AGN			
Commissions	−0.99 (−1.67–0.31)	0.005	−0.207
Omissions	−0.89 (−1.64–0.13)	0.022	−0.213
Latency	2.55 (−3.37-8.46)	0.395	0.029
Affective bias	9.93 (1.61–18.24)	0.02	0.334

For DMS, there was no change in number of correct responses (β = 0.33, 95% CI: −0.64 to 1.31, *p* =.50) or latency (β = −97.66, 95% CI: −221.98 to 26.66, *p* =.12) from pre to post treatment. For **RVP**, participants improved on number of correct hits (β = 3.18, 95% CI: 1.82 to 4.55, *p* <.001), number of misses (β = −2.61, 95% CI: −3.43 to −1.79, *p* <.001) and mean latency (β = −13.54, 95% CI: −26.48 to −0.6, *p* =.04) after treatment, whereas no difference was seen on number of false positives (β = 0.08, 95% CI: −0.41 to 0.57, *p* =.74).

For OTS, participants improved on number of problems solved on first choice (β = 0.45, 95% CI: 0.02 to 0.88, *p* =.04), but not mean choices to correct (β = −0.02, 95% CI: −0.05 to 0.01, *p* =.12) or mean latency (β = −1678.04, 95% CI: −3445.95 to 89.88, *p* =.06) after treatment.

For SSP, there was no change in span length (β = −0.01, 95% CI: −0.31 to 0.29, *p* =.95), number of attempts (β = −0.26, 95% CI: −0.72 to 0.21, *p* =.28), errors (β = −1.04, 95% CI: −2.69 to 0.62, *p* =.22) or latency (β = −328.06, 95% CI: −878.33 to 222.22, *p* =.24).

For AGN, participants had fewer commissions (β = −0.99, 95% CI: −1.67 to −0.31, *p* =.01) and omissions (β = −0.89, 95% CI: −1.64 to −0.13, *p* =.02) and a positive effect on affective bias (β = 9.93, 95% CI 1.61 to 18.24, *p* =.02) after treatment, but there was no difference in latency (β = 2.55, 95% CI: −3.37 to 8.46, *p* =.40).

### Associations between changes in ISI/MADRS-S and changes in cognitive function

Associations between change in cognitive function from pre to post treatment and change in ISI and MADRS-S scores were investigated using linear regression, and results are summarized in Table 5. There were no significant associations between changes in any of the cognitive tasks and changes on the ISI or MADRS-S scores.

**Table 5:** Associations between change in cognitive variable and change in MADRS-S/ISI during treatment with either CBT-I or CBT-D (*N* = 170). Insdep = Insomnia and depression study. Ins = Insomnia study with adaptive treatment. DMS = Delayed matching to Sample, RVP = Rapid visual Processing, OTS = One touch stockings of Cambridge, SSP = Spatial span Task, AGN = Affective go/No go

Variable	MADRS-S change			ISI change		
	Estimate (95% CI)	Standardised correlation	p	Estimate (95% CI)	Standardised correlation	p
DMS						
Number of correct responses	−0.007 (−0.13-0.12)	−0.01	0.917	−0.01 (−0.16-0.14)	−0.01	0.894
Mean latency	−3.351 (−20-13.3)	−0.04	0.691	−5.01 (−25.22-15.2)	−0.04	0.624
RVP						
Correct hits	0.059 (−0.13-0.25)	0.06	0.544	0.112 (−0.13-0.35)	0.08	0.352
Number of misses	0.062 (−0.06-0.18)	0.09	0.311	0.07 (−0.08-0.22)	0.08	0.352
False positives	0.022 (−0.04-0.09)	0.06	0.498	0.045 (−0.03-0.12)	0.1	0.26
Mean latency	−0.901 (−2.71-0.91)	−0.09	0.326	1.018 (−1.23-3.27)	0.08	0.372
OTS						
Number of problems solved on first choice	0.006 (−0.05-0.06)	0.02	0.831	0.016 (−0.05-0.08)	0.04	0.637
Mean choices to correct	0 (0–0)	0	0.991	−0.001 (−0.01-0)	−0.02	0.814
Mean latency	174.555 (−58.2-407.31)	0.13	0.14	240.09 (−42.25-522.43)	0.15	0.095
SSP						
Span length	−0.024 (−0.06-0.01)	−0.11	0.219	−0.023 (−0.07-0.02)	−0.09	0.336
Number of attempts	−0.043 (−0.11-0.02)	−0.12	0.177	−0.036 (−0.11-0.04)	−0.08	0.354
Errors	−0.134 (−0.36-0.09)	−0.11	0.235	−0.124 (−0.4-0.15)	−0.08	0.37
Latency	−38.337 (−113.81-37.13)	−0.09	0.317	−73.303 (−164.55-17.94)	−0.14	0.114
AGN						
Commissions	−0.033 (−0.12-0.06)	−0.06	0.477	−0.021 (−0.14-0.09)	−0.03	0.716
Omissions	−0.001 (−0.1-0.1)	0	0.991	−0.066 (−0.19-0.06)	−0.09	0.298
Latency	0.367 (−0.46-1.19)	0.08	0.379	0.504 (−0.52-1.53)	0.09	0.331
Affective bias	0.028 (−1.15-1.21)	0	0.963	0.403 (−1.04-1.84)	0.05	0.581

### Associations between baseline cognitive function and treatment effect (changes in ISI)

Associations between baseline cognitive values and treatment effect, measured as change in ISI from baseline to post treatment, was investigated using linear regression and is presented in Table 6. The only baseline variable that was associated with treatment effect was number of omissions in the AGN, which was positively correlated with a larger change in ISI from pre to post treatment (β = 0.202, 95% CI: 0.08 to 0.32, *p* =.00).

**Table 6:** Prediction of baseline cognitive variables and change in ISI in patients receiving CBT-I (*N* = 124). Insdep = Insomnia and depression study. Ins = Insomnia study with adaptive treatment, DMS = Delayed matching to Sample, RVP = Rapid visual Processing, OTS = One touch stockings of Cambridge, SSP = Spatial span Task, AGN = Affective go/No go

Variable	Crude model		Partly adjusted model^1^		Fully adjusted model^2^	
	Estimate (95% CI)	*p*	Estimate (95% CI)	*p*	Estimate (95% CI)	*p*
DMS						
Number of correct responses	−0.043 (−0.39-0.3)	0.804	−0.029 (−0.39-0.34)	0.874	−0.064 (−0.39-0.26)	0.698
Mean latency	0 (0–0)	0.622	0 (0–0)	0.487	0 (0–0)	0.502
RVP						
Correct hits	−0.042 (−0.19-0.1)	0.562	−0.071 (−0.22-0.08)	0.359	−0.042 (−0.18-0.1)	0.545
Number of misses	0.041 (−0.16-0.24)	0.685	0.091 (−0.13-0.31)	0.405	0.033 (−0.16-0.23)	0.74
False positives	−0.355 (−0.83-0.12)	0.144	−0.346 (−0.86-0.17)	0.187	−0.202 (−0.67-0.27)	0.399
Mean latency	0.002 (−0.01-0.01)	0.67	0.003 (−0.01-0.02)	0.624	−0.003 (−0.01-0.01)	0.588
OTS						
Number of problems solved on first choice	−0.041 (−0.42-0.34)	0.831	−0.037 (−0.43-0.36)	0.855	−0.112 (−0.47-0.24)	0.534
Mean choices to correct	−1.096 (−7.28-5.08)	0.726	−0.932 (−7.55-5.69)	0.781	−0.064 (−6.02-5.89)	0.983
Mean latency	0 (0–0)	0.817	0 (0–0)	0.548	0 (0–0)	0.882
SSP						
Span length	−0.424 (−1.14-0.29)	0.244	−0.412 (−1.24-0.42)	0.328	−0.515 (−1.26-0.23)	0.173
Number of attempts	−0.359 (−0.82-0.11)	0.13	−0.33 (−0.85-0.19)	0.207	−0.323 (−0.79-0.14)	0.17
Errors	−0.044 (−0.2-0.11)	0.576	−0.024 (−0.19-0.14)	0.771	−0.043 (−0.19-0.11)	0.564
Latency	0 (0–0)	0.741	0 (0–0)	0.937	0 (0–0)	0.66
AGN						
Commissions	0.116 (−0.09-0.32)	0.261	0.141 (−0.07-0.35)	0.185	0.09 (−0.1-0.28)	0.351
Omissions	0.148 (0.02–0.28)	0.025	0.18 (0.04–0.32)	0.011	0.202 (0.08–0.32)	0.001
Latency	−0.003 (−0.02-0.01)	0.716	−0.005 (−0.02-0.01)	0.575	−0.005 (−0.02-0.01)	0.527
Affective bias	−0.018 (−0.05-0.01)	0.236	−0.022 (−0.05-0.01)	0.176	−0.015 (−0.04-0.01)	0.3

^1^Adjusted for Age, Sex, Education, depression and NART

^2^Adjusted Age, Sex, Education, and NART, ISI score and use of sleep medication

## Discussion

This study examined cognitive performance of individuals with insomnia, with and without comorbid depression, and assessed changes in objective cognitive function following internet-delivered therapist-supported CBT-I. Overall, we found limited evidence for a strong association between baseline depression comorbid with insomnia and cognitive performance across most domains. However, in the spatial span task, individuals with comorbid depression were both slower to respond and demonstrated shorter memory spans compared to those without depression. This finding suggests that depression comorbid with insomnia may have domain-specific effects on cognitive performance, particularly in tasks requiring spatial working memory and processing speed.

Our findings diverge from those of a recent meta-analysis assessing cognitive performance using the CANTAB battery in depressed patients compared to healthy controls, which found no significant differences in spatial span performance across eight studies involving 583 participants [68]. In the same meta-analysis, the most sensitive CANTAB measures for detecting cognitive differences between depressed and non-depressed individuals were RVP and OTS, while the AGN task was not included [68]. The discrepancy between our results and the meta-analytic findings may be attributable to the fact that all participants in our sample had insomnia, a condition that could independently impair cognitive function, thereby potentially modifying the cognitive profile typically observed in depression.

Following CBT-I treatment, participants across groups exhibited improvements on some cognitive tasks. Notably, statistically significant gains were observed in sustained attention (as measured by RVP), executive function and planning (as assessed by OTS), working memory, and affective information processing (via AGN). For RVP the effect sizes where of medium size (Cohen’s d around 0.5 depending on outcome), whereas the other effect sizes were small. The change in OTS is consistent with a previous meta-analysis reporting the OTS to detect improvements following therapeutic interventions [68]. The same meta-analysis could not demonstrate the same for RVP [68]. While these changes may partially reflect practice effects or non-specific time effects, the consistency across some cognitive domains might support the possibility of treatment-related cognitive enhancement. On the other hand, Kyle et al. [39] conducted a large RCT involving 410 participants with insomnia. That study reported treatment effects in favor of digital CBT-I for subjective cognitive failures, sleep parameters, and mood, but did not observe improvements in objective cognitive performance, in contrast to our study. In relation to affective processing, Tamm and colleagues [69] reported no change in affective facial recognition following CBT-I despite symptomatic improvement but also self-reported emotional regulation and mood, highlighting that not all domains of emotional processing might be affected uniformly, and that subjectively reported and objectively measures do not necessarily capture the same effects. One can argue that subjective measures of cognition are at least as clinically valid as objective measures with varying ecological validity [70], and adding concurrent subjective and objective measures to further studies would be a valuable contribution. Nevertheless, research on objective measures of cognitive functioning will inform the field and possibly lead to new research questions, if not clinically applicable conclusions. The positive change in the AGN task is more in line with studies of antidepressants but also behavioral activation, suggesting an association between depression treatment response and reduced negative bias [71–73]. Interestingly, baseline omissions on the AGN task, but not any other cognitive variable, predicted greater improvement in insomnia symptoms following treatment. This is partly in contrast to one previously published study suggesting that worse results on the Wisconsin Card Sort Task, but no other cognitive task predicted better treatment outcome in CBT for depression [74].

Despite improvements in cognitive performance from pre to post treatment, we found no statistically significant associations between changes in cognitive function and reductions in depressive symptoms (MADRS-S) or insomnia severity (ISI). This could suggest that the observed cognitive gains may occur independently of symptomatic improvement but could also indicate that changes are not specifically related to the treatment but rather to unspecific time effects. However, the mechanisms by which CBT-I might improve cognitive function remain an area of active inquiry. Sleep is essential for neurocognitive functioning, particularly for memory consolidation, attention, and executive processing [26, 75–77]. By improving sleep continuity and efficiency, CBT-I may enhance the restorative properties of sleep, thereby facilitating improvements in attention, working memory, and planning. These mechanisms merit further investigation, particularly in light of the limited association between symptomatic improvement and cognitive change observed in this study. From a clinical perspective, the findings underscore the potential of CBT-I not only to alleviate sleep and mood symptoms but possibly also to enhance cognitive functioning in individuals with insomnia.

This study has several limitations that should be considered when interpreting the findings. First, the absence of a control group for longitudinal analyses and lack of randomization limits the ability to infer causality, and the possibility of practice or time effects cannot be excluded. Second, although the sample included patients with and without depression, group differences and potential confounding variables, such as medication or other psychiatric symptoms such as anxiety, were not controlled experimentally. There were also no objective measures of sleep. Third, the generalizability of the findings may be constrained by the sample size and demographic characteristics, and additional studies are needed to confirm these results in larger, more diverse populations. We investigated effects across two treatment groups, on one hand potentially increasing generalizability, on the other hand introducing a possible confounder in terms of treatment length. Finally, while we employed validated tasks to assess cognitive function, the interpretation of domain-specific effects should be made cautiously, given the complexity of overlapping cognitive processes.

In conclusion, this study suggests that CBT-I may be associated with improvements in objective cognitive performance across some domains, including attention, working memory, executive function, and emotional processing. Although depression was not broadly associated with cognitive impairment in this sample, specific deficits in spatial span performance were observed among patients with comorbid depression. Future research should employ randomized controlled designs and explore the long-term cognitive effects of CBT-I. 

## Supplementary Information

Supplementary Material 1.

## Data Availability

The datasets generated and/or analyzed during the current study are not publicly available due privacy reasons and GDPR legislations but are available from the corresponding author on reasonable request.

## Abbreviations

RCT: Randomized Controlled Trial
AGN: Affective Go/No-go
CANTAB: Cambridge Neuropsychological Test Automated Battery
CBT: Cognitive Behavioral Therapy
CBT-I: Cognitive Behavioral Therapy for Insomnia
DMS: Delayed Matching to Sample
ISI: Insomnia Severity Index
MADRS-S: Montgomery Åsberg Depression Rating Scale – self rated
MOT: Motor Screening
NART: National Adult Reading Test
OTS: One Touch Stockings of Cambridge
RVP: Rapid Visual Processing
SSP: Spatial Span
