# Supplementary material for: Objectively measured cognitive function in insomnia patients with and without comorbid depression treated with cognitive behavioral therapy for insomnia
Source: BMC Psychiatry. 2025 Oct 2;25:916. doi: 10.1186/s12888-025-07460-5 (PMC12490117; doi:10.1186/s12888-025-07460-5)

Appendix

**Suppl. Fig 1 Study Flowcharts for the two clinical trials**

**
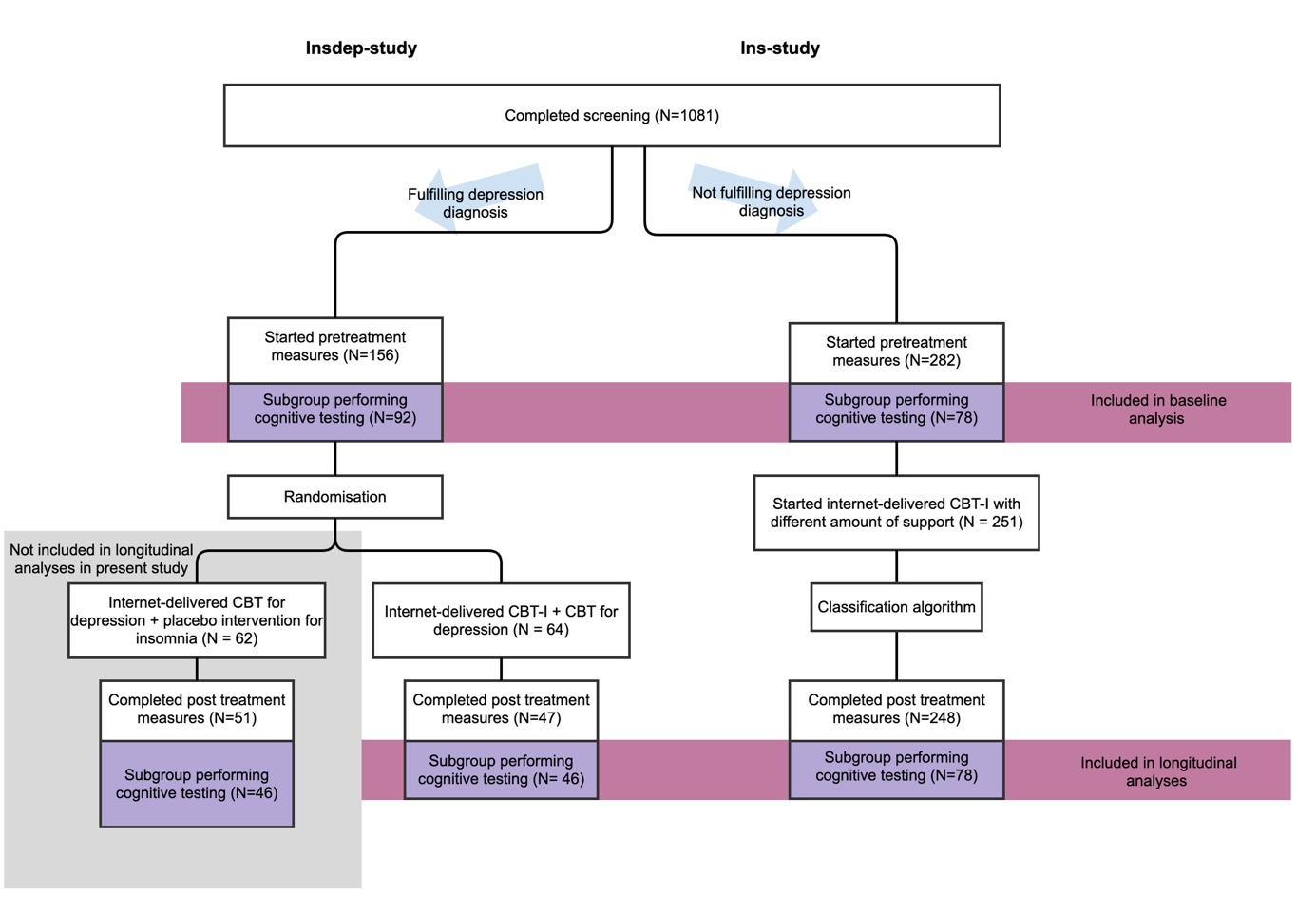
**

**Suppl. Table 1. Content of the insomnia treatment in the Ins-study**

| **Module** | **Content** |
| --- | --- |
| 1 | How the platform works, facts about sleep. What is sleep, what is it for? How much sleep do we need, and what consequences does sleep have? Is there such a thing as morning and evening people? What is insomnia? |
| 2 | More about insomnia – how do we get insomnia and why does it not remit spontaneously? Introduction to cognitive behavioral therapy. Information about sleep medication and creating a cessation plan (optional) |
| 3 | Learning the difference between feeling tired and actually being sleepy/sleep deprived. Learning about the myths about sleep that may exacerbate insomnia. |
| 4 | Introduction to sleep restriction and getting started |
| 5 | Introduction to stimulus control and getting started |
| 6 | Routines for getting sunlight and exercise during the day and creating bed-time routines for consistency and winding down before bed |
| 7 | Acceptance, mindfulness, attitudes and expectations on sleep |
| 8 | Cognitive reappraisal, focused on thoughts, rumination and worry about sleep |
| 9 | Information and exercises about sleep hygiene such as avoiding caffeine later in the day, not drinking alcohol close to bedtime, keeping your bedroom cool and dark, and not having heavy meals at night |
| 10 | Some extra mindfulness materials and repetition of acceptance and cognitive reappraisal |
| 11 | Summary and relapse prevention |

**Suppl. Table 2. Content of the combination treatment in the Insdep-study**

| Module | Content |
| --- | --- |
| 1 | Information about the treatment. Psychoeducation about depression and insomnia. Circadian rhythm. CBT principles. |
| 2 | Brief information on sleep hygiene. Description of SRT and BTC+SC with instructions. Introduction to activity planning. |
| 3 | Start using chosen sleep strategy (SRT or BTC+SC). Lear about and start using stimulus control. Information on napping. Handling of sleep medications. Visualization strategies. |
| 4 | Activity planning / behavioral activation (BA). Value compass. Problem solving method. |
| 5 | Focus: Continued use of sleep strategies and BA. Also: Tips on how to handled difficulties with the methods. Tips on managing fatigue and sleepiness. Psychoeducation methods for handling anxiety and stress. Tips for engaging in physical exercise. Relaxation techniques. Going-to-bed-routines. |
| 6 | Continue to use sleep and BA strategies. Psychoeducation about acceptance and cognitive reappraisal: negative thoughts, rumination, worrying, choosing valued behavior despite neg. thoughts and evaluate consequences (behavioral experiments), focus shifting. |
| 7 | Continue to use sleep and BA strategies. Thought distancing: defining and nuancing problematic terms and beliefs, thinking traps. |
| 8 | Focus: continued work with introduced methods. Problem solving around adherence to treatment. Also: psychoeducation about an exercises in mindfulness. |
| 9 | Follow-up on goals. Re-evaluate value compass. Tips on how to handle adversity and setbacks. |

SRT = sleep restriction therapy, BTC+SC = bed-time consistency with optional addition of sleep compression, BA = behavioral activation

**Suppl. Table 3. Sleep medication and antidepressants reported at pre treatment (numbers of participants (%)**

| **Medication** | **Ins-study** | **Ins-dep** |
| --- | --- | --- |
| Zolpidem | 22 (28.21%) | 12 (13.04%) |
| Zopiclone | 17 (21.79%) | 13 (14.13%) |
| Zaleplon | 1 (1.28%) | 0 |
| Oxazepam | 4 (5.13%) | 2 (2.17%) |
| Diazepam | 0 | 1 (1.09%) |
| Propiomazine | 13 (16.67%) | 8 (8.70%) |
| Hydroxyzine | 4 (5.13%) | 7 (7.61%) |
| Promethazine | 2 (2.56%) | 3 (3.26%) |
| Cetirizine | 1 (1.28%) | 0 |
| Melatonin | 4 (5.13%) | 5 (5.43%) |
| Alimemazine | 0 | 3 (3.26%) |
| Valerian | 4 (5.13%) | 0 |
| Mirtazapine | 2 (2.56%) | 1 (1.09%) |
| Citalopram | 3 (3.85%) | 2 (2.17%) |
| Sertraline | 2 (2.56%) | 9 (9.78%) |
| Fluoxetine | 0 | 2 (2.17%) |
| Escitalopram | 1 (1.28%) | 0 |
| Bupropion | 0 | 1 (1.09%) |
| Moclobemide | 0 | 1 (1.09%) |
| Reboxetine | 0 | 1 (1.09%) |
| Venlafaxine | 0 | 1 (1.09%) |

**Suppl. fig 2-4. Visualization of pre to post changes in cognitive variables divided by study.** Thin lines represent individuals, and thicker lines represent group means


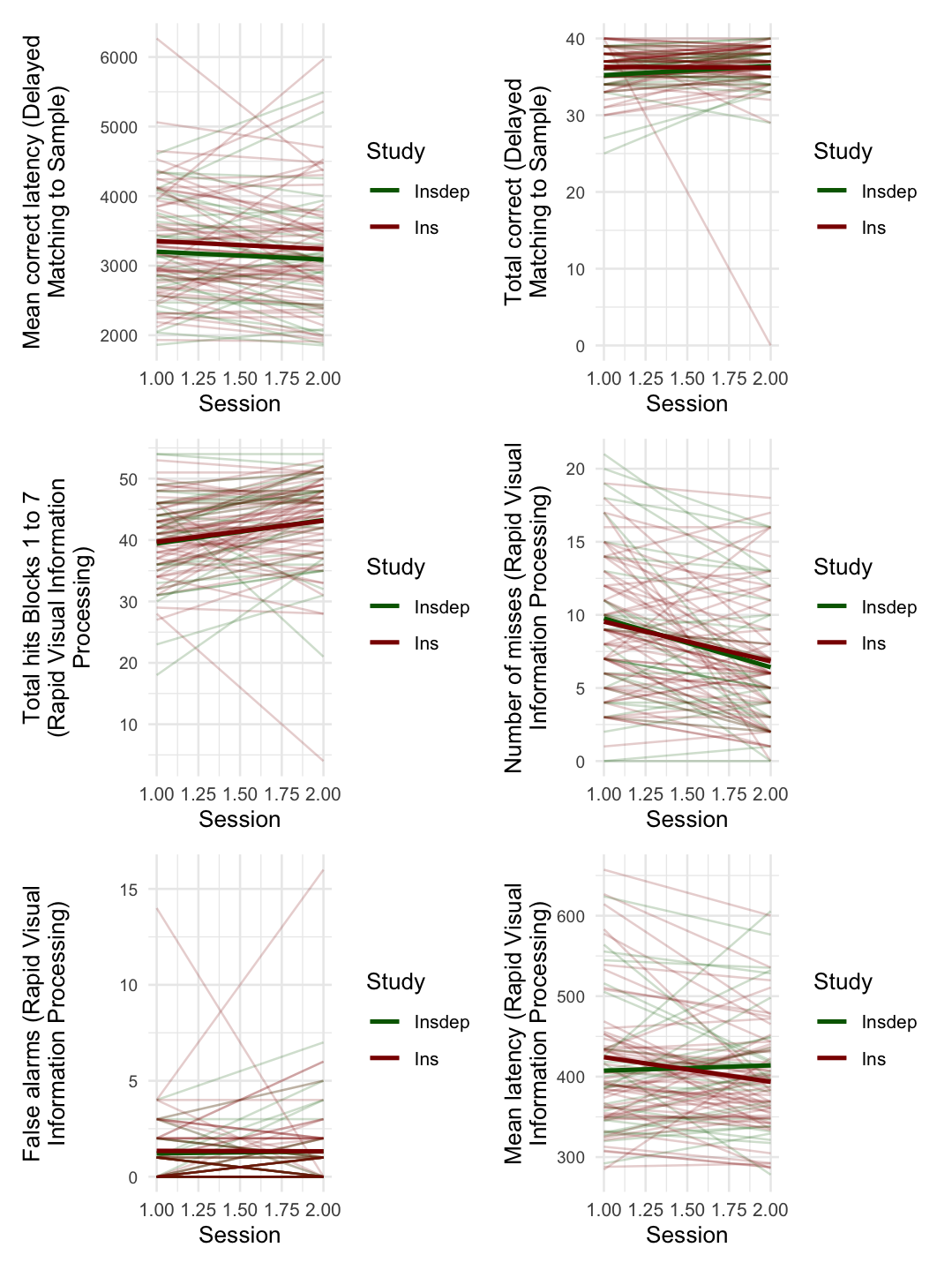


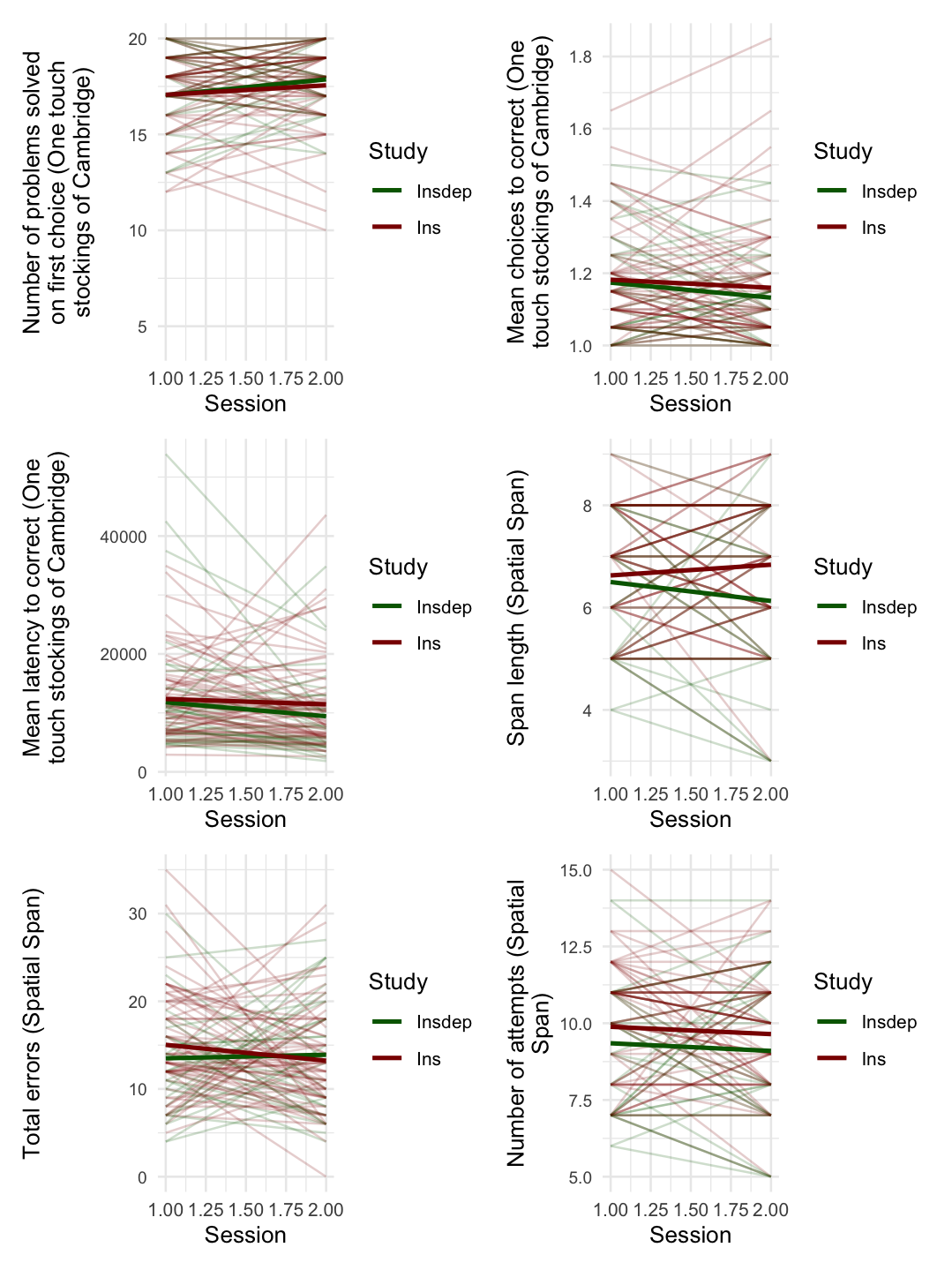


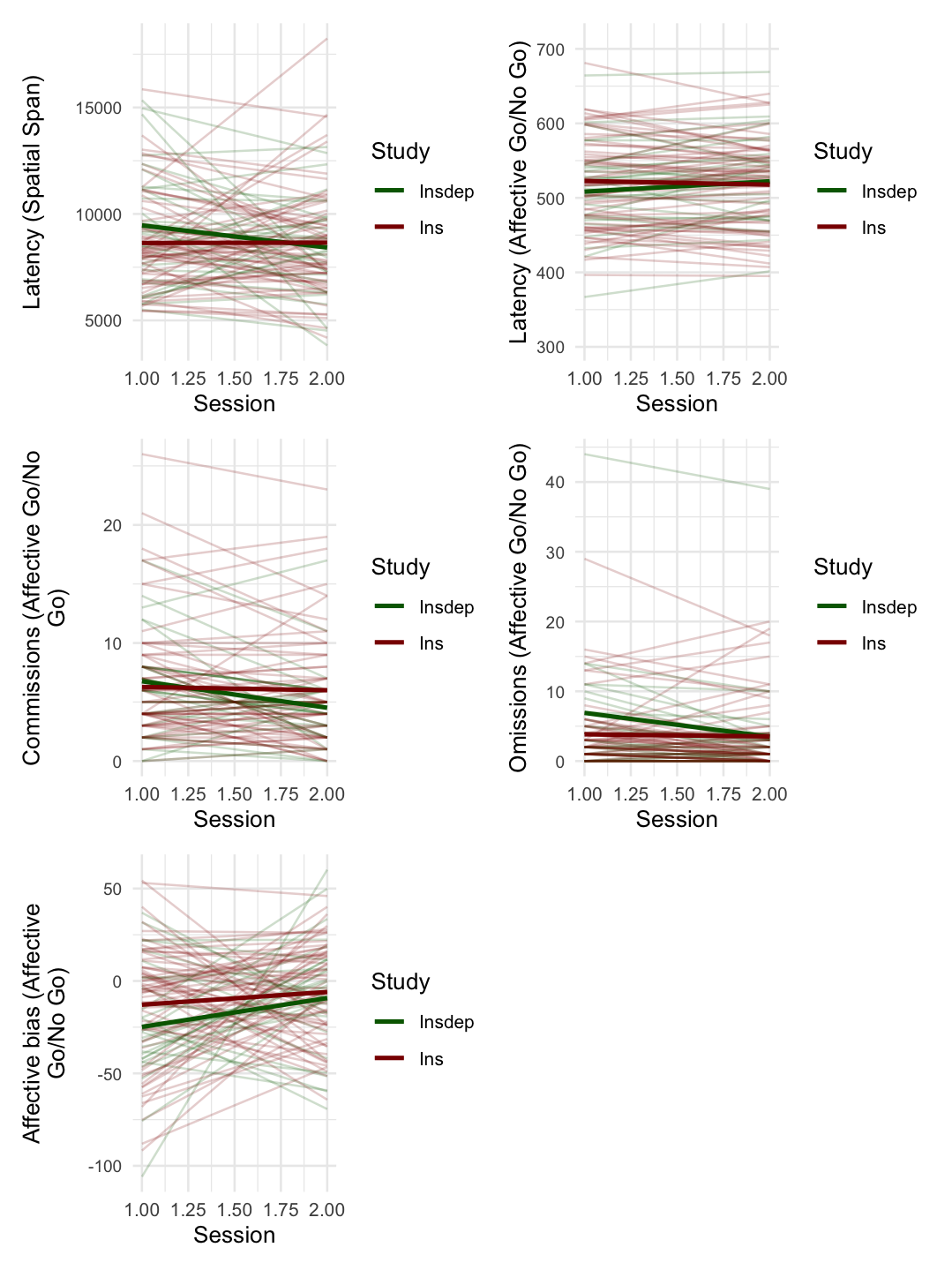

Supplement: Supplementary file 1 — Supplementary Material 1. [file 12888_2025_7460_MOESM1_ESM.docx]
